# Red cell distribution width to albumin ratio (RAR) as a prognostic marker for mortality in critical care patients: a large-scale retrospective cohort study

**DOI:** 10.1186/s40001-025-02903-x

**Published:** 2025-07-23

**Authors:** Chaoyuan Jin, Ruijinlin Hao, Xingxing Ren, Jie Shen

**Affiliations:** 1 Center of Emergency & Intensive Care Unit, Jinshan Hospital, Fudan University, Shanghai, 201508 China; 2 Medical Center of Chemical Injury, Jinshan Hospital, Fudan University, Shanghai, 201508 China; 3 Medical Research Center for Chemical Injury, Emergency and Critical Care of Chemical Injury, Jinshan Hospital, Fudan University, Shanghai, 201508 China; 4 Department of Anesthesiology, The First Affiliated Hospital With Nanjing Medical University, Nanjing, 210029 China; 5 Department of Endocrinology and Metabolism, Zhongshan Hospital, Fudan University, Shanghai, 200032 China

**Keywords:** Red cell distribution width to albumin ratio, Intensive care, Mortality, Prognostic marker, Medical Information Mart for Intensive Care IV database

## Abstract

**Background:**

Prognosis of critically ill patients is strongly correlated with nutritional and inflammatory states. This study aimed to evaluate the potential of the red cell distribution width to albumin ratio (RAR) as a mortality predictor in ICU patients, focusing on its prognostic value in diverse patient subgroups.

**Methods:**

In this retrospective cohort study, 24,568 adult ICU patients were analyzed. RAR was calculated upon admission, and the relationship between RAR and 28-day mortality was assessed using Cox proportional hazards modeling and restricted cubic spline analysis to explore nonlinearity. Receiver operating characteristic (ROC) analysis assessed predictive performance, and subgroup analyses examined the prognostic strength of RAR across age groups, disease severity, and required interventions.

**Results:**

The predictive power of RAR for 28-day mortality was moderate (AUC = 0.66, 95% CI 0.65–0.67). Multivariate analysis showed that higher RAR values were associated with a higher risk of death (adjusted HR = 1.06, 95% CI 1.05–1.07, P < 0.001) and a significant nonlinear relationship (*P* < 0.001). Subgroup analyses showed that the prognostic value of RAR was higher in patients aged ≥ 65 years and in patients with a SOFA score of ≥ 6. Combining RAR with SOFA scores improved predictive accuracy (AUC = 0.74, 95% CI 0.72–0.76), suggesting that RAR has the potential to be an adjunct to mortality risk assessment in the ICU setting.

**Conclusions:**

Although RAR demonstrates only moderate predictive ability on its own (AUC = 0.66), it significantly enhances prognostic accuracy when combined with SOFA scores (AUC 0.74), suggesting its role as a complementary rather than independent prognostic tool in ICU risk stratification.

## Introduction

Despite advances in critical care practice and improved treatment strategies, predicting intensive care unit (ICU) patient mortality remains a critical yet challenging task [1, 2]. The current scoring systems such as Acute Physiology and Chronic Health Evaluation (APACHE), Sequential Organ Failure Assessment (SOFA), and Simplified Acute Physiology Score (SAPS) aid in risk assessment; however, they can be complex and may require multiple inputs that are not always readily available in time-sensitive clinical settings. Therefore, simpler, cost-effective, and widely available prognostic markers are needed to complement existing tools, enabling more effective risk stratification and better-informed clinical decision-making [3–5]. Identifying such markers is particularly important in intensive care, where rapid patient assessment is essential for managing limited resources effectively.

Growing evidence underscores the critical role of inflammation and nutrition in determining outcomes in ICU patients [6, 7]. Among hematologic markers, red cell distribution width (RDW) has gained attention as a potentially useful predictor in critical illness. RDW, a measure of the variability in red blood cell volume, often increases in response to systemic inflammation and oxidative stress, both of which are prevalent in critically ill patients [8]. Elevated RDW levels have been consistently associated with worse clinical outcomes in various conditions, including sepsis, heart failure, and chronic obstructive pulmonary disease (COPD), suggesting RDW as a marker of underlying pathophysiological processes in these patients [9, 10].

Serum albumin, traditionally regarded as an indicator of nutritional status, also decreases significantly during critical illness, often in response to inflammation. Low albumin levels have been linked to poorer outcomes and complications, underscoring the interconnectedness of nutritional and inflammatory pathways in critically ill patients [11]. However, the broader applicability of RAR in diverse ICU populations has not been thoroughly investigated, and its role in mortality prediction across different ICU subgroups remains unclear.

The RDW-to-albumin ratio (RAR) has emerged as a novel composite marker that may offer additional prognostic insight in this context [12, 13]. Early studies have evaluated RAR in specific contexts, such as myocardial infarction, stroke, heart failure, burn surgery, severe acute pancreatitis, chronic kidney disease, acute kidney injury, and sepsis, with initial findings supporting its role as a mortality predictor [14–22].

In this study, we aimed to fill this gap by analyzing a large ICU cohort from the Medical Information Mart for Intensive Care IV (MIMIC-IV) version 3.0 database. Our objectives were to (1) characterize the association between RAR and mortality in ICU patients, (2) identify subgroups within the ICU population where RAR demonstrates the highest prognostic value, and (3) assess the additional predictive value that RAR may provide when used alongside established risk scores, such as APACHE or SOFA [23–25].

A clearer understanding of RAR’s role could enhance clinicians' ability to perform risk stratification in critically ill patients, potentially improving both patient outcomes and resource allocation in the ICU setting. Importantly, because RDW and albumin are routinely measured in clinical laboratories, RAR could be an accessible, low-cost tool for evaluating patient prognosis in real-time, facilitating its integration into standard care practices.

## Methods

### Study design and population

This retrospective cohort study utilized data from the MIMIC-IV v3.0 database. The authors completed the required training course (certification number: 65849805) and obtained authorization to access the database through PhysioNet. The study protocol was exempt from formal review by the Institutional Review Boards of MIT (Cambridge, MA) and Beth Israel Deaconess Medical Center (Boston, MA), with waiver of informed consent due to the retrospective nature of the study using de-identified data.

We analyzed adult patients (aged 18–90 years) admitted to ICU between 2008 and 2022. Exclusion criteria included (1) missing RDW or albumin values at admission; (2) ICU stays less than 24 h; and (3) age < 18 or > 90 years. For patients with multiple ICU admissions during the study period, only data from their first admission were included in the analysis to maintain statistical independence of observations.

### Data collection

Demographic data included age, gender, and weight. Clinical variables encompassed vital signs, mechanical ventilation status, vasopressor use, renal replacement, ECMO, blood transfusion, and comorbidities. Laboratory parameters, including complete blood count, biochemical indices, and coagulation profiles, were collected from the first tests performed within 24 h of ICU admission. Disease severity was assessed using the Sequential Organ Failure Assessment (SOFA) score.

### Outcomes

The primary outcome was all-cause mortality within 28 days after ICU admission. Survival status was determined through in-hospital records available in the MIMIC-IV database.

### Statistical analysis

Continuous variables were presented as median (interquartile range) and compared using the Kruskal–Wallis test, while categorical variables were expressed as frequencies (percentages) and compared using chi-square tests. The predictive performance of RAR and other clinical parameters was assessed through receiver-operating characteristic (ROC) curve analysis, with area under the curve (AUC) values and 95% confidence intervals calculated. To evaluate the association between RAR and outcomes, Cox proportional hazards models were constructed with three levels of adjustment: unadjusted (Model 1), adjusted for age (≥ 65 vs < 65 years), sex (male vs female), and SOFA score (≥ 6 vs < 6) (Model 2), and further adjusted for mechanical ventilation, vasopressor use, and lactate (Model 3). Restricted cubic spline analyses were performed to examine potential nonlinear relationships. Interaction testing and subgroup analyses were conducted to assess effect modification across different patient populations. Missing data were handled using multiple imputation when appropriate. Statistical significance was set at *P* < 0.05 (two-sided). Data processing and analysis were performed using R version 4.4.0 (2024–04-24).

## Results

### Baseline characteristics

**Fig. 1:**
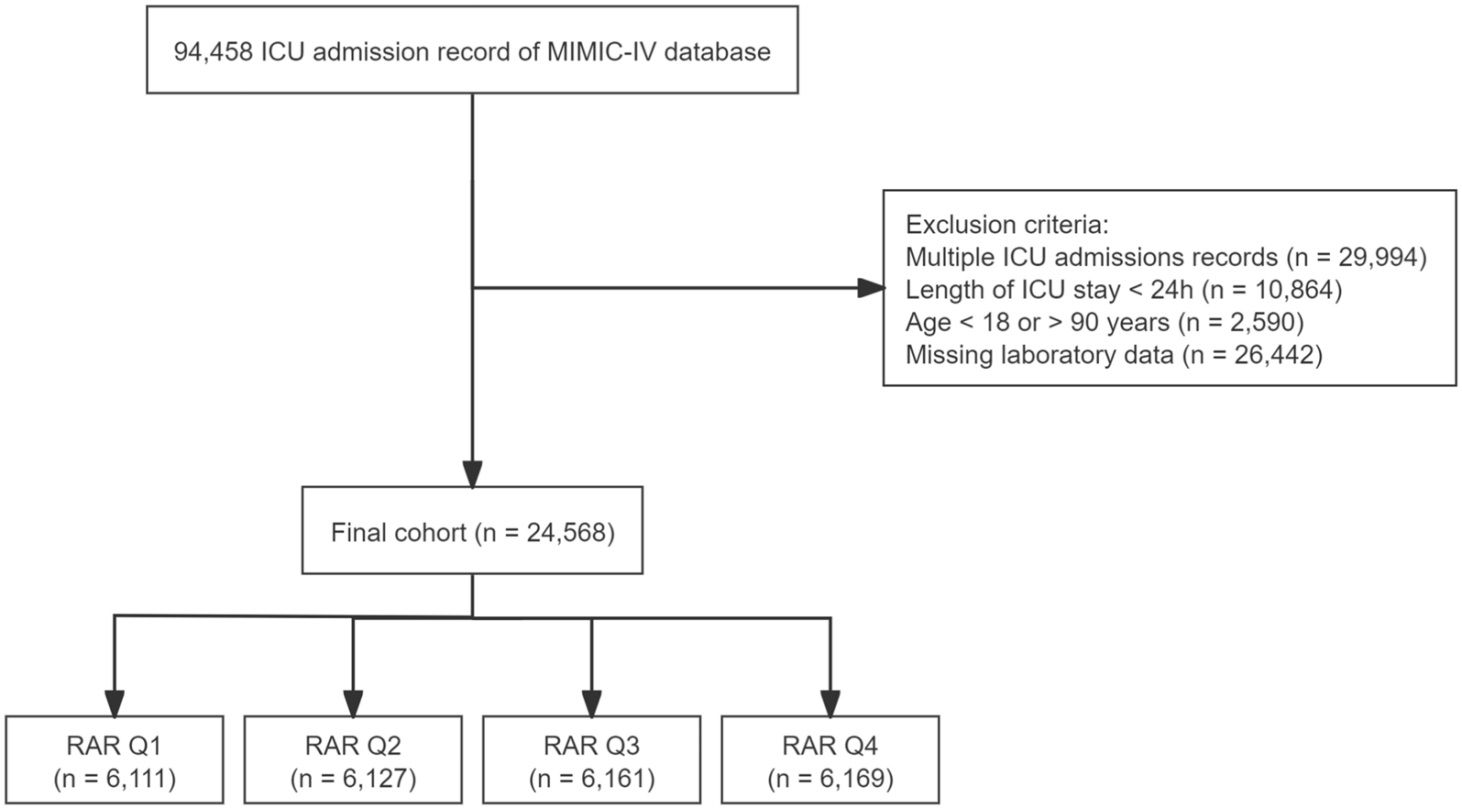
Selection of the study population from the MIMIC-IV database

In this study, we analyzed a cohort of 24,568 ICU patients, classified into quartiles based on RAR values (Fig. 1). The cohort’s median age was 66 years (IQR: 54–76). Baseline characteristics significantly varied across RAR quartiles, with patients in the higher quartiles showing elevated median heart rates, respiratory rates, and Sequential Organ Failure Assessment (SOFA) scores. Additionally, these patients exhibited lower albumin levels and higher red cell distribution width (RDW) values, indicating more severe clinical presentations (all *P* < 0.001) (Table 1).﻿﻿

**Table 1:** Patient demographics and baseline characteristics

Variables Median (IQR)	Total (*n* = 24,568)	Quartile 1 (*n* = 6111)	Quartile 2 (*n* = 6127)	Quartile 3 (*n* = 6161)	Quartile 4 (*n* = 6169)	*P*
RAR	4.91 (4.05, 6.12)	3.61 (3.33,3.84)	4.46 (4.25,4.68)	5.44 (5.15,5.73)	7.19 (6.57,8.24)	** < 0.001**
Age (years)	66.00 (54.00, 76.00)	63.00 (50.00,74.00)	67.00 (55.00,77.00)	67.00 (57.00,77.00)	66.00 (56.00,76.00)	** < 0.001**
Weight (kg)	79.30 (66.50, 94.60)	79.80 (67.10,94.47)	80.00 (67.22,95.30)	79.30 (66.60,95.50)	77.60 (65.10,92.80)	** < 0.001**
Heart rate	89.00 (76.00, 105.00)	84.00 (72.00,97.00)	88.00 (75.00,102.00)	91.00 (77.00,107.00)	95.00 (81.00,111.00)	** < 0.001**
Respiratory rate	19.00 (16.00, 23.00)	18.00 (15.00,22.00)	19.00 (16.00,23.00)	19.00 (16.00,24.00)	20.00 (16.00,24.00)	** < 0.001**
SBP (mmHg)	120.00 (105.00, 138.00)	129.00 (113.00,145.00)	122.00 (106.00,140.00)	118.00 (103.00,136.00)	113.00 (98.00,130.00)	** < 0.001**
SpO2 (%)	98.00 (95.00, 100.00)	98.00 (96.00,100.00)	98.00 (95.00,100.00)	98.00 (95.00,100.00)	98.00 (95.00,100.00)	** < 0.001**
SOFA	5.00 (3.00, 9.00)	4.00 (2.00,6.00)	5.00 (3.00,8.00)	6.00 (4.00,9.00)	7.00 (4.00,11.00)	** < 0.001**
RDW (%)	14.70 (13.50, 16.50)	13.30 (12.80,13.90)	14.30 (13.50,15.20)	15.40 (14.30,16.80)	17.20 (15.60,19.30)	** < 0.001**
ALB (g/dL)	3.10 (2.60, 3.50)	3.80 (3.50,4.00)	3.20 (3.00,3.40)	2.80 (2.60,3.10)	2.30 (2.10,2.60)	** < 0.001**
WBC (10^9^/L)	10.90 (7.60, 15.60)	10.30 (7.70,13.90)	10.90 (7.80,14.80)	11.00 (7.50,15.90)	11.90 (7.50,18.30)	** < 0.001**
HGB (g/dL)	10.50 (8.90, 12.30)	12.30 (11.00,13.60)	10.80 (9.40,12.30)	9.90 (8.60,11.40)	9.10 (7.90,10.50)	** < 0.001**
PLT (10^9^/L)	190.00 (129.75, 261.00)	204.00 (159.00,258.50)	189.00 (135.00,254.00)	180.00 (118.00,262.00)	175.00 (100.00,273.00)	** < 0.001**
Creatinine (mg/dL)	1.00 (0.70, 1.60)	0.90 (0.70,1.20)	1.00 (0.70,1.60)	1.10 (0.80,1.80)	1.20 (0.80,2.10)	** < 0.001**
Sodium (mmol/L)	138.00 (135.00, 141.00)	139.00 (136.00,141.00)	138.00 (136.00,141.00)	138.00 (135.00,141.00)	138.00 (134.00,141.00)	** < 0.001**
Potassium(mmol/L)	4.10 (3.70, 4.60)	4.00 (3.70,4.40)	4.10 (3.70,4.60)	4.20 (3.70,4.70)	4.10 (3.70,4.70)	** < 0.001**
Glucose (mg/dL)	128.00 (104.00, 167.00)	126.00 (104.00,160.00)	130.00 (107.00,169.00)	131.00 (106.00,172.00)	125.00 (100.00,166.00)	** < 0.001**
LAC (mmol/L)	1.80 (1.40, 2.40)	1.80 (1.40,1.90)	1.80 (1.30,2.20)	1.80 (1.30,2.50)	1.80 (1.40,3.00)	** < 0.001**
AST (IU/L)	39.00 (23.00, 81.00)	37.00 (22.00,60.00)	39.00 (24.00,76.00)	39.00 (24.00,89.00)	42.00 (24.00,101.00)	** < 0.001**
INR (ratio)	1.30 (1.10, 1.60)	1.20 (1.10,1.30)	1.30 (1.10,1.50)	1.30 (1.20,1.60)	1.40 (1.30,1.80)	** < 0.001**
Gender						** < 0.001**
Female	10,484 (42.67)	2355 (38.54)	2533 (41.34)	2777 (45.07)	2819 (45.70)	
Male	14,084 (57.33)	3756 (61.46)	3594 (58.66)	3384 (54.93)	3350 (54.30)	
Mechanical ventilation						** < 0.001**
No	11,388 (46.35)	3258 (53.31)	2750 (44.88)	2749 (44.62)	2631 (42.65)	
Yes	13,180 (53.65)	2853 (46.69)	3377 (55.12)	3412 (55.38)	3538 (57.35)	
Vasopressor use						** < 0.001**
No	17,442 (70.99)	5196 (85.03)	4535 (74.02)	4129 (67.02)	3582 (58.06)	
Yes	7126 (29.01)	915 (14.97)	1592 (25.98)	2032 (32.98)	2587 (41.94)	
Hypertension						** < 0.001**
No	15,216 (61.93)	3311 (54.18)	3660 (59.74)	3992 (64.79)	4253 (68.94)	
Yes	9352 (38.07)	2800 (45.82)	2467 (40.26)	2169 (35.21)	1916 (31.06)	
Diabetes						** < 0.001**
No	17,575 (71.54)	4701 (76.93)	4320 (70.51)	4236 (68.76)	4318 (70.00)	
Yes	6993 (28.46)	1410 (23.07)	1807 (29.49)	1925 (31.24)	1851 (30.00)	
Heart failure						** < 0.001**
No	17,661 (71.89)	4902 (80.22)	4234 (69.10)	4175 (67.76)	4350 (70.51)	
Yes	6907 (28.11)	1209 (19.78)	1893 (30.90)	1986 (32.24)	1819 (29.49)	
28-day all-cause mortality						** < 0.001**
No	19,871 (80.88)	5527 (90.44)	5229 (85.34)	4901 (79.55)	4214 (68.31)	
Yes	4697 (19.12)	584 (9.56)	898 (14.66)	1260 (20.45)	1955 (31.69)	

Patients in the highest RAR quartile (Q4) had a higher likelihood of requiring mechanical ventilation (57.35%) and vasopressor support (41.94%) than those in the lowest quartile (Q1) (46.69% and 14.97%, respectively) (*P* < 0.001).

### Association between RAR and 28-day mortality

**Fig. 2:**
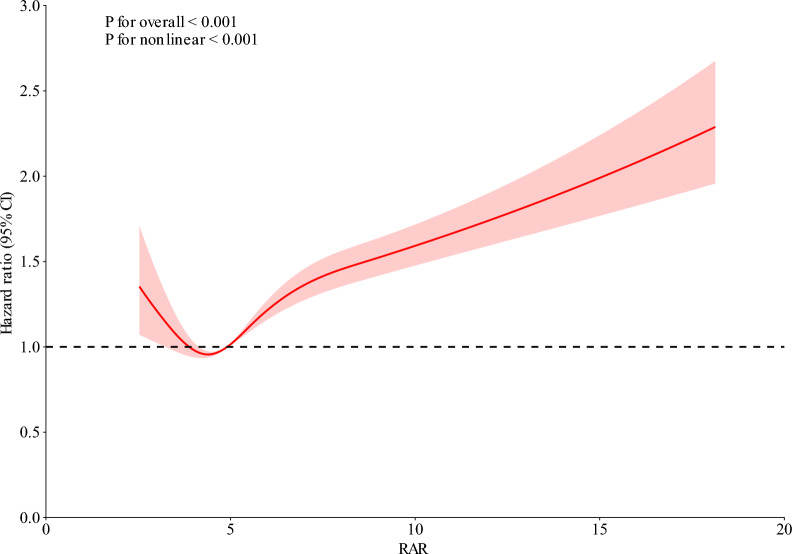
Restricted cubic spline (RCS) analysis of 28-day all-cause mortality by RAR. RCS analysis showing adjusted hazard ratios (HR) for 28-day mortality at varying RAR levels, with 95% confidence intervals (CI). The vertical line indicates the RAR level with the lowest mortality risk; the horizontal line represents an HR of 1.0. HR, hazard ratio; CI, confidence interval

**Fig. 3:**
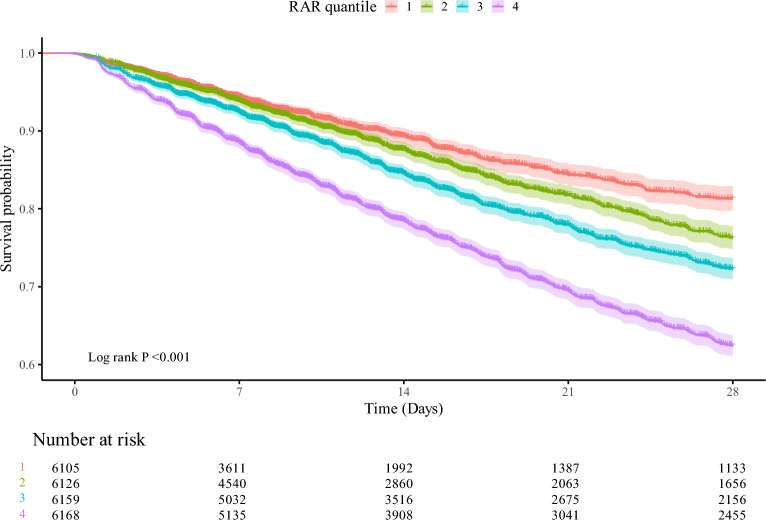
Kaplan–Meier survival curves for 28-day mortality by RAR quartiles. Kaplan–Meier survival curves showing 28-day mortality across RAR quartiles. Survival probability declines with higher RAR quartiles, with statistically significant differences confirmed by the log-rank test

Restricted cubic spline analysis revealed a significant nonlinear relationship between RAR and 28-day mortality, with a sharp increase in mortality risk observed as RAR values exceeded approximately 5.0 (*P* for nonlinearity < 0.001) (Fig. 2). The inflection point at RAR ≈ 5.0 was identified through visual inspection of the spline curve, corresponding to the point where the mortality risk trajectory began to increase more sharply. Kaplan–Meier survival curves confirmed this dose–response relationship, showing 28-day survival rates decreasing from 90.44% in Q1 to 68.31% in Q4 (log-rank *P* < 0.001) (Fig. 3).

Cox proportional hazards models confirmed that higher RAR independently predicted increased mortality risk. In the fully adjusted model, each unit increase in RAR was associated with a 6% rise in 28-day mortality risk (HR = 1.06, 95% CI 1.05–1.07, *P* < 0.001) after controlling for age, gender, SOFA score, mechanical ventilation, vasopressor use, and lactate levels (Table 2). Patients in the highest RAR quartile (Q4) had a 52% higher mortality risk compared to Q1 (adjusted HR = 1.52, 95% CI 1.39–1.68, *P* < 0.001).

**Table 2:** Association between RAR quartiles and 28-day mortality in cox proportional hazard models

Characteristics	Model 1 HR (95% CI)	Model 2 HR (95% CI)	Model 3 HR (95% CI)
RAR quartiles			
Q1 (Reference)	1.00	1.00	1.00
Q2	1.22 (1.10–1.35)***	1.02 (0.92–1.13)	1.00 (0.90–1.11)
Q3	1.50 (1.36–1.65)***	1.18 (1.07–1.31)***	1.13 (1.03–1.25)*
Q4	2.18 (1.99–2.39)***	1.66 (1.51–1.83)***	1.52 (1.39–1.68)***
Demographic characteristics			
Age ≥ 65 years	–	1.53 (1.44–1.62)***	1.56 (1.47–1.66)***
Male sex	–	0.95 (0.89–1.00)	0.94 (0.89–1.00)
Clinical parameters			
SOFA score ≥ 6	–	2.36 (2.21–2.52)***	1.63 (1.51–1.76)***
Mechanical ventilation	–	–	1.28 (1.19–1.37)***
Vasopressor use	–	–	1.46 (1.37–1.56)***
Lactate (per mmol/L)	–	–	1.11 (1.10–1.12)***

Cox proportional hazard models assessing the association between RAR quartiles and 28-day mortality

Model 1 is unadjusted

Model 2 is adjusted for age, sex, and SOFA score

Model 3 is further adjusted for mechanical ventilation, vasopressor use, and lactate levels

Hazard ratios (HR) and 95% confidence intervals (CI) are presented

*P < 0.05; **P < 0.01; ***P < 0.001

### Predictive value of RAR compared with other biomarkers

**Fig. 4:**
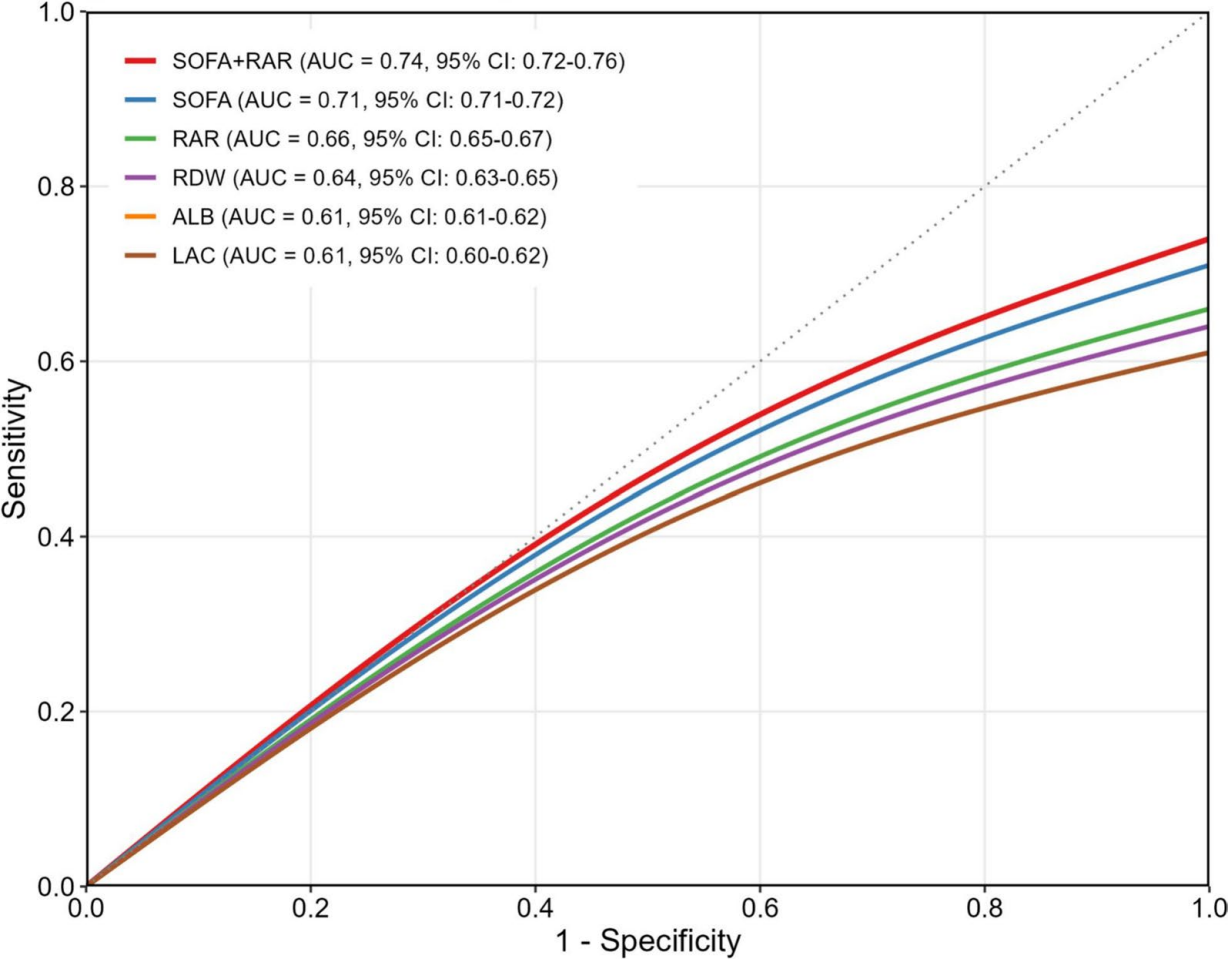
Receiver operating characteristic (ROC) curve analysis for 28-day mortality prediction. ROC curve analysis comparing the predictive value of RAR and other biomarkers for 28-day mortality. The area under the curve (AUC) for each marker demonstrates the ability of RAR to predict mortality relative to other ICU biomarkers and scoring systems

Receiver-operating characteristic (ROC) curve analysis compared RAR with other commonly used ICU biomarkers and scores, including SOFA, RDW, albumin (ALB), and lactate (LAC), for their ability to predict mortality. The combination of SOFA and RAR yielded the highest area under the curve (AUC = 0.74, 95% CI 0.72–0.76), outperforming SOFA alone (AUC = 0.71, 95% CI 0.71–0.72), RAR alone (AUC = 0.66, 95% CI 0.65–0.67), RDW (AUC = 0.64, 95% CI 0.63–0.65), ALB (AUC = 0.61, 95% CI 0.61–0.62), and LAC (AUC = 0.61, 95% CI 0.60–0.62) (Fig. 4).

### Subgroup analyses

**Fig. 5:**
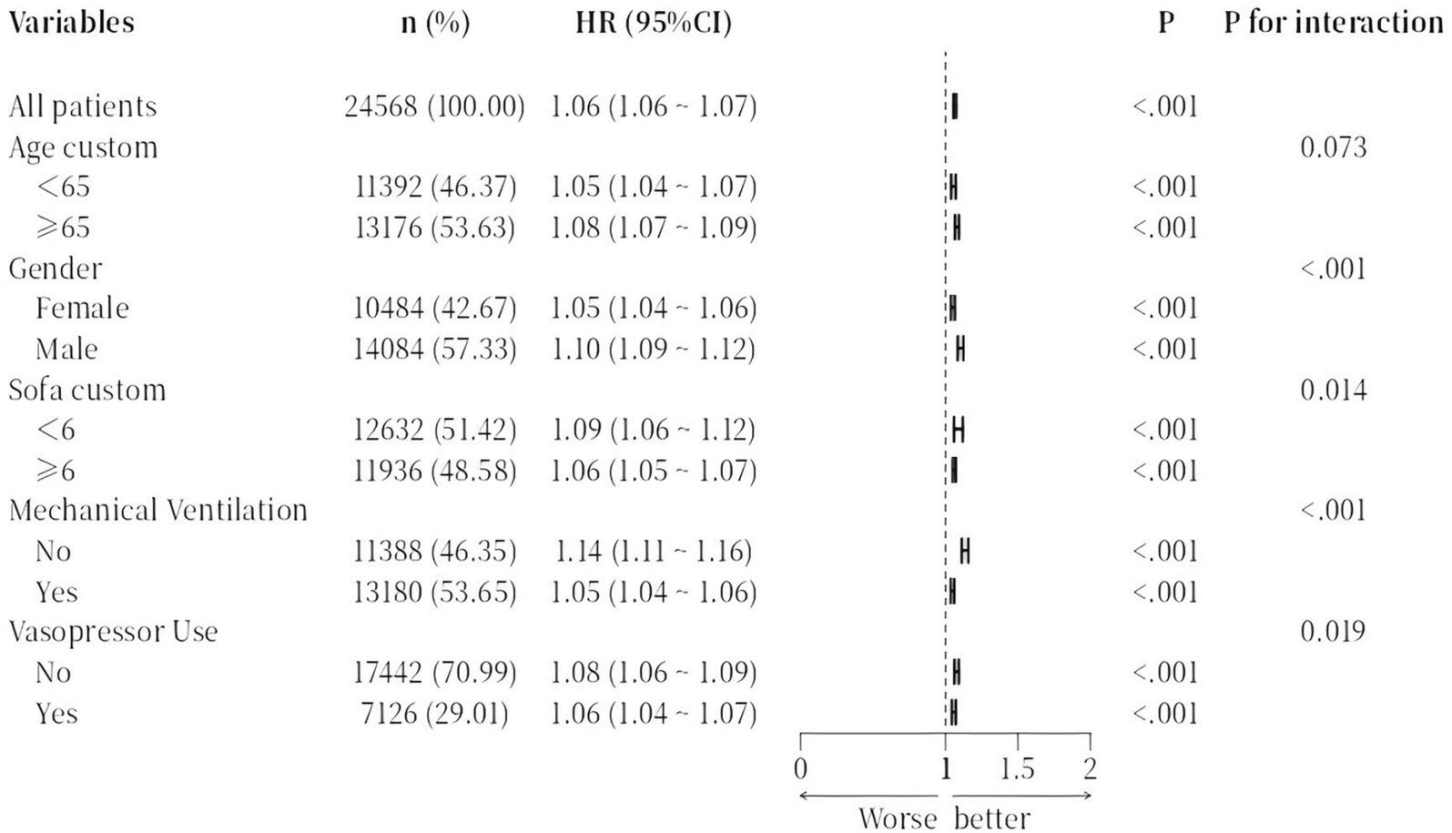
Subgroup analysis Forest plot for 28-day all-cause mortality. Forest plot displaying the adjusted hazard ratios for 28-day all-cause mortality across various patient subgroups. Adjustments include age, sex, SOFA scores. The plot highlights potential interactions between RAR and subgroup characteristics, with subgroup-specific HRs and 95% CIs shown

The prognostic value of RAR remained consistent across various subgroups, including age, gender, and clinical severity (Fig. 5). In patients aged ≥ 65 years, each unit increase in RAR was linked to a greater mortality risk (HR = 1.08, 95% CI 1.07–1.10) as compared to patients younger than 65 years (HR = 1.04, 95% CI 1.03–1.06), with a significant interaction observed (*P* for interaction = 0.003). In addition, patients with a SOFA score ≥ 6 showed a stronger association between RAR and mortality than those with a SOFA score < 6 (*P* for interaction < 0.001), as detailed in Fig. 5. These findings suggest that RAR has heightened prognostic significance in older patients and those with more severe illness.

## Discussion

This study investigated the role of the red cell distribution width to albumin ratio (RAR) as a predictor of 28-day mortality in critically ill patients, revealing its significant prognostic value. RAR was found to be independently associated with mortality, with a nonlinear relationship suggesting that risk escalates sharply when RAR exceeds approximately 5.0 [15, 26]. This nonlinear association highlights the potential of RAR as a simple yet effective tool for identifying patients at higher risk, enabling earlier clinical interventions.

RAR provides unique insights into critical illness by combining two critical aspects of patient status: systemic inflammation and nutritional health [26]. Elevated RDW reflects oxidative stress and inflammation, conditions commonly observed in ICU patients and associated with adverse outcomes. Simultaneously, hypoalbuminemia serves as a marker of malnutrition and acute inflammatory responses. By integrating these two parameters, RAR captures a composite picture of the patient’s physiological state, offering a more holistic marker than either variable alone. This dual utility underscores its relevance in the ICU, where rapid and accurate risk stratification is essential.

Our findings also highlight the importance of RAR in specific subgroups [18, 20]. Older patients (≥ 65 years) and those with severe organ dysfunction, as indicated by higher SOFA scores, demonstrated a stronger association between elevated RAR and mortality. These populations often exhibit a higher inflammatory burden and greater vulnerability to nutritional deficits, making RAR a particularly relevant marker. Interestingly, the integration of RAR into established scoring systems such as SOFA significantly improved predictive performance, with a combined AUC of 0.74. This suggests that RAR may enhance traditional scoring systems, providing added precision without requiring additional complex measurements.

Compared with other prognostic markers commonly used in the ICU, RAR offers several practical advantages [14, 21]. It is derived from routine laboratory parameters, making it widely available and cost-effective. Furthermore, its simplicity enables clinicians to incorporate it into standard care workflows without additional training or resources. This accessibility is particularly valuable in resource-limited settings, where advanced scoring systems may not always be feasible. The nonlinear relationship observed also implies that RAR may help highlight patients at elevated risk, although further studies are needed to define clinically actionable thresholds. Despite these advantages, the moderate AUC of 0.66 indicates that RAR's optimal use is as a complementary tool in combination with established scoring systems like SOFA, where it can meaningfully enhance predictive accuracy from 0.71 to 0.74, rather than serving as a standalone prognostic marker.

Despite these strengths, several limitations of this study warrant discussion. The retrospective design, although allowing for the analysis of a large cohort, introduces potential biases, including unmeasured confounders [23]. In addition, as a single-center analysis, our findings require external validation before RAR can be recommended for widespread clinical use. The reliance on the MIMIC-IV database, while providing access to a diverse patient population, may limit generalizability due to institutional practices and patient demographics. We also did not stratify patients by primary admission diagnosis, which limits understanding of RAR's performance in specific disease states. Another limitation is the single time-point measurement of RAR at admission, which does not account for potential changes in the ratio over time. Dynamic fluctuations in RAR during the ICU stay may provide additional prognostic information, and this remains an important area for future research. Finally, the moderate discriminatory ability of RAR as a standalone marker (AUC 0.66) clearly indicates it should be used as a complementary tool to enhance existing scoring systems rather than replacing them.

In summary, RAR is a promising prognostic tool for critically ill patients, offering a balance of simplicity, accessibility, and predictive accuracy. It holds particular value for high-risk subgroups, such as the elderly and those with severe organ dysfunction, and complements established scoring systems to refine mortality risk prediction. Future prospective studies are needed to validate these findings across diverse populations, evaluate the impact of serial RAR measurements, and explore its utility in guiding treatment decisions. With further research, RAR could become an integral part of ICU risk assessment, improving both patient management and resource allocation.

## Conclusion

RAR is a practical complementary biomarker that enhances mortality prediction in ICU patients when combined with established scoring systems. While demonstrating moderate standalone predictive ability, its true value lies in augmenting existing tools like SOFA scores. The simplicity and accessibility of RAR, derived from routine laboratory parameters, make it a cost-effective adjunct for risk stratification, particularly beneficial for elderly patients and those with severe organ dysfunction. Prospective multicenter validation is needed. Future research should explore disease-specific applications, serial RAR measurements, and development of RAR-integrated risk prediction models. With further validation, RAR could become a valuable component of ICU risk assessment, improving both patient management and resource allocation.

## Data Availability

The dataset supporting the conclusions of this article is available in the MIMIC-IV repository, accessible at https://mimic.mit.edu/. Access to the MIMIC-IV database requires successful completion of a required training course and approval from PhysioNet. The study utilizes publicly accessible, de-identified data available to authorized researchers.

## Abbreviations

ALB: Albumin
APACHE: Acute Physiology and Chronic Health Evaluation
AST: Aspartate aminotransferase
AUC: Area under the curve
CI: Confidence interval
COPD: Chronic obstructive pulmonary disease
ECMO: Extracorporeal membrane oxygenation
HGB: Hemoglobin
HR: Hazard ratio
ICU: Intensive care unit
INR: International normalized ratio
IQR: Interquartile range
LAC: Lactate
MIMIC-IV: Medical Information Mart for Intensive Care IV
MIT: Massachusetts Institute of Technology
PLT: Platelet count
RAR: Red cell distribution width to albumin ratio
RCS: Restricted cubic spline
RDW: Red cell distribution width
ROC: Receiver-operating characteristic
SAPS: Simplified Acute Physiology Score
SBP: Systolic blood pressure
SOFA: Sequential Organ Failure Assessment
SpO2: Blood oxygen saturation
WBC: White blood cell count
